# Preoperative Mouthwash in Subjects with Different Periodontal Status: A Randomised Controlled Clinical Trial

**DOI:** 10.3290/j.ohpd.a44308

**Published:** 2020-07-04

**Authors:** Priscila de Macedo Máximo, Sheila Cavalca Cortelli, Davi Romeiro Aquino, Taís Browne de Miranda, Fernando Oliveira Costa, José Roberto Cortelli

**Affiliations:** a PhD Student, School of Dentistry, Department of Periodontology, University of Taubaté,Taubaté, SP, Brazil. Performed the clinical collection, participated in the study design, wrote the manuscript.; b Professor, Department of Dentistry, Periodontics Research Division, University of Taubaté, Taubaté, SP, Brazil. Participated in the study design and revision of the manuscript.; c Professor, Department of Dentistry, Periodontics Research Division, University of Taubaté, Taubaté, SP, Brazil. Performed the clinical collection and statistical analysis.; d PhD Student, School of Dentistry, Department of Periodontology, University of Taubaté,Taubaté, SP, Brazil. Performed the laboratory analysis, and article formatting.; e Professor, School of Dentistry, Department of Periodontology, Federal University of Minas Gerais, Belo Horizonte, MG, Brazil. Participated in the study design and revision of the manuscript.; f Professor, Department of Dentistry, Periodontics Research Division, University of Taubaté, Taubaté, SP, Brazil. Participated in the study design, wrote and revised the manuscript, supervised the experiments.

**Keywords:** anti-infective agents, mouthwashes, periodontal diseases, single dose

## Abstract

**Purpose:**

The effects of three preoperative mouthwashes on salivary bacterial levels were evaluated and compared between subjects with differing periodontal status.

**Materials and Methods:**

Based on periodontal parameters, periodontally healthy individuals (n = 60) and those with gingivitis (n = 60) and periodontitis (n = 60) were randomly assigned to a single preoperative dose of chlorhexidine (CHX), essential oils (EO), cetylpyridinium chloride (CPC) or negative control mouthwashes. Saliva samples were collected between 8:00 and 11:00 a.m., before and after a single-dose rinse with the respective mouthwash. Total bacterial load and levels of *Porphyromonas gingivalis, Tannerella forsythia, Treponema denticola* and *Streptococcus oralis* were determined by qPCR. Data were statistically analysed using paired t- and Student’s t-tests (p < 0.05).

**Results:**

CHX, EO and CPC showed greater antimicrobial efficacy than did the negative control. CHX [1226445.53] and EO [1743639.38] provided greater reductions in comparison to both CPC [106302.96] and negative control [37852.46]). CHX provided greater reductions of simultaneous levels of Pg [106326.00], Td [3335841] and Tf [61557.47] in the healthy group, as did EO in the diseased groups. CPC provided the greatest reduction [3775319.36] in the periodontitis group.

**Conclusion:**

Periodontal status influenced the antimicrobial efficacy of preoperative mouthwashes. Therefore, periodontal status should be taken into consideration by clinicians. The antimicrobial efficacy differed among the agents tested. CHX and EO showed the greatest efficacy. The recognition of periodontal condition by clinicians is mandatory to select the most effective preoperative mouthwash.

Most procedures performed by dental professionals have the potential for creating contaminated aerosols and splatter, which create a risk of disease transmission to clinicians and patients. Therefore, preoperative mouthwashes are among the strategies for controlling dental cross-infection. Before dental procedures, single doses of mouthwashes have been used for different purposes, such as bacterial reduction in dental aerosols,^21^ reduction of intra-oral infection,^29^ and even reduction of bacteremia.^2^ Logothetis et al^19^ showed that both EO and CHX reduced bacterial contamination in aerosols, while Reddy et al^22^ demonstrated that tempered CHX was more effective than non-tempered CHX when used as a preoperative rinse. In addition, a CPC, zinc lactate, and sodium fluoride preoperative mouthwash effectively reduced viable bacteria in aerosols produced by ultrasonic scaler.^23^

The microbial shifts that take place during the transition from a clinically healthy to a diseased periodontium are well known. Quantitative changes in bacterial levels as well as qualitative changes in biofilm composition have been observed.^9,32^ In fact, Diaz et al^9^ suggested that some bacterial species act as modulators during transitional phases. Belstrom et al^4^ found higher frequencies and levels of eight bacterial taxa and four bacterial clusters among individuals with periodontitis in comparison to controls. Further, through an experimental gingivitis model, Lee et al^17^ successfully identified microbial signatures related to gingival inflammation. Therefore, it is reasonable to expect some influence of periodontal status on the antimicrobial efficacy of commercial active ingredients used as preoperative mouthwashes.

Although preoperative mouthwashes tend to be more effective in comparison to water or negative controls, their effectiveness is not uniform. However, studies to date have not been designed to elucidate which factors are responsible for this variability. Therefore, despite discrepancies, there is a lack of evidence on how oral local factors could influence the effectiveness of preoperative mouthwashes.

The present study hypothesised that in the presence of periodontal disease, greater bacterial reductions would take place in comparison to periodontal health. To test this hypothesis, salivary bacterial levels before and after preoperative mouthwashes were compared among periodontally healthy individuals and those with gingivitis and periodontitis.

## Materials and Methods

### Study Population

Participants included in the present study were recruited from the University of Taubate, SP, Brazil from September 2016 to March 2017. All subjects signed an informed consent form that was previously approved by the Institutional Committee on Research Involving Human Subjects (protocol 37231214.9.0000.5501).

To be included in the study, the female and male subjects had to have at least 20 natural teeth in the oral cavity, and be in good general health between the ages of 18 and 69. Smokers and nonsmokers were included in this study. Individuals were excluded if they were: (I) poorly managed diabetics, (II) immunosuppressed, (III) pregnant/lactating women, (IV) experiencing hormonal changes, (V) users of extensive fixed prostheses/orthodontic appliances, (VI) individuals submitted to local or systemic antibiotic therapy in the last six months, (VII) those who had received periodontal treatment in the last 12 months. Finally, individuals who made regular use of any mouthwash were also excluded.

**Fig 1:**
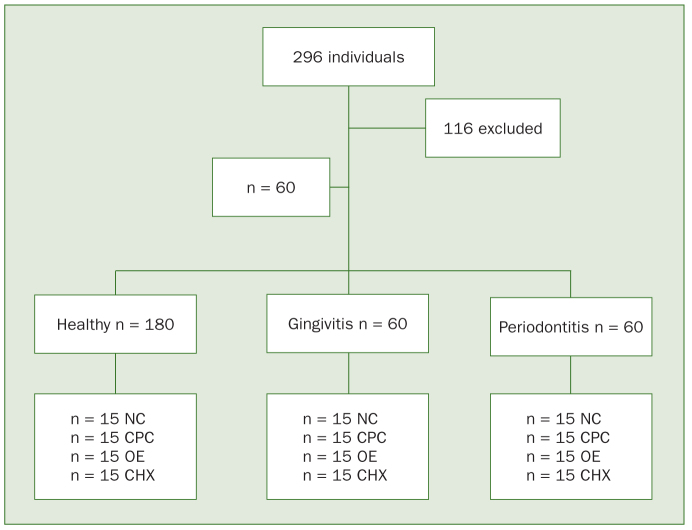
Flow chart of study design. NC: negative control; CPC: cetylpyridinium chloride; EO: essential oils; CHX: chlorhexidine.

The desired sample size of 60 subjects per group was based on the results previously reported in the literature^26^ and was calculated to provide 80% power. Three main groups were formed based on periodontal status: healthy (n = 60); gingivitis (n = 60); periodontitis (n = 60). The flow chart in Fig 1 shows study design (Fig 1).

### Clinical Examination

Clinical measurements were used to establish periodontal condition. Plaque (PI)^27^ and gingival indices (GI),^18^ periodontal probing depth (PPD) and clinical attachment level (CAL) were taken on all teeth except for third molars, using a millimeter periodontal Williams probe (Hu-Friedy; Chicago, IL, USA). One trained and calibrated examiner, blinded to type of treatment, conducted all clinical measurements. Baseline data analysis was performed to determine whether intra-examiner reliability was calibrated. Using kappa statistics (κ) for the categorical clinical measurement variables, such as periodontal probing depth and clinical attachment level, the standard error of these measurements was calculated. The examiner’s clinical measurement technique was considered calibrated if the standard error for the measurements was ≤ 0.8 and the κ-value ranged between 0.8 and 0.95. The reproducibility of the intra-examiner measurements was recalculated prior to the final clinical exams.^8^

Gingivitis was diagnosed under the following conditions: PPD ≤ 3 mm and presence of gingival redness and bleeding on probing (BOP) in ≥ 10% of sites.^7^ Periodontitis stage 1 was present when subjects had PPD ≤ 4 mm and CAL between 1 and 2 mm; grade A was determined when the percentage of bone loss/age < 0.25 and there was evidence of no bone loss over 5 years.^20^ Participant were considered periodontally healthy when they showed BOP < 10% and an absence of CAL.^7^

### Experimental Groups

Participants in each periodontal status group (healthy, gingivitis or periodontitis) were randomly distributed by the closed-envelope system into four subgroups (n = 15) according to mouthwash formula:

- CPC: 0.07% cetylpyridinium chloride (Oral B Pro-Health, Proctor and Gamble; Cincinnati, OH, USA)
- EO: essential oils: menthol 42%, thymol 0.064%, eucalyptol 0.092% and methyl salicylate 0.06% (Listerine Cool Mint, Johnson and Johnson; New Brunswick, NJ, USA)
- CHX: chlorhexidine 0.12% (PerioGard, Colgate-Palmolive; New York NY, USA);
- NC: negative control solution: hydroalcohol 0.5% (FarmaVale; Lorena, Brazil).

In all subgroups, participants rinsed with 20 ml of the designated solution for 30 s under professional supervision.

### Saliva Sampling and Microbial Analysis

Two milliliters of unstimulated saliva samples were collected in sterile 15 ml falcon tubes from the participants between 08:00 and 11:00 a.m., before and immediately after the use of the mouthwash or negative control solution. No food or drink was permitted for two hours before collection. During sample collection, the participants remained in a seated position, with their head tilted forward (approximately 45 degrees), in a quiet, well-ventilated room.

The samples were centrifuged (10,000×g for 10 min at 4°C) and 500 µl of the supernatant was transferred to a 1.5 ml tube, which was centrifuged (10,000×g for 3 min at 25°C) and the supernatant discarded, yielding a pellet. Genomic DNA (gDNA) was extracted from the pellet and purified using PureLink Genomic DNA Mini Kit (Life Technologies; Carlsbad, CA, USA) according to the manufacturer’s specifications. The quantification of total bacterial cells *Treponema denticola, Porphyromonas gingivalis, Tannerella forsythia* and *S. oralis* was carried out by real-time quantitative PCR (qPCR) using the TaqMan assay (TaqMan Universal PCR Master Mix II, Life Technologies) with a specific set of primers/probes (Table 1) in an ABI 7500 Fast Real Time PCR System (Life Technologies) following the manufacturer’s instructions in 20 µl reactions. The qPCR conditions were: 50°C for 2 min, 95°C for 10 min, 40 cycles of 95°C for 15 min and 60°C for 1 min. The target of the bacteria’s primers and probes are the 16S gene.

**Table 1:** Description of primers and probes used in the quantitative real time PCR assays

Microorganisms		Primers and probes
Gram positive and negative bacteria	Total bacterial load	F: TGGAGCATGTGGTTTAATTCGA
		R: TGCGGGACTTAACCCAACA
		Probe: CACGAGCTGACGACAAGCCATGCA
Gram negative bacteria	*Treponema denticola*	F: CCGAATGTGCTCAATTACATAAAGGT
		R: GATACCCATCGTTGCCTTGGT
		Probe: ATGGGCCCGCGTCCCATTAGC
	*Porphyromonas gingivalis*	F: ACCTTACCCGGGATTGAAATG
		R: CAACCATGCAGCACCTACATAGAA
		Probe: ATGACTGATGGTGAAAACCGTCTTCCCTTC
	*Tanerella forsythia*	F: AGCGATGGTAGCAATACCTGTC
		R: TTCGCCGGGTTATCCCTC
		Probe: CACGGGTGAGTAACG
Gram positive bacterium	*Streptococcus oralis*	F: TTGGCTCAATTCCCTTTGAC
		R: GTCCAAACAAGCCACCACTT
		Probe: ACAACATATCAACAGGCGCA

The absolute quantification of the target organisms was determined by plotting the cycle threshold (Ct) value obtained from each clinical sample against a standard curve generated with a known concentration of gDNA from ATCC strains provided by Fiocruz (Oswaldo Cruz Foundation, Instituto Nacional de Controle de Qualidade em Saúde [INCQS], Rio de Janeiro, Brazil) in 1:10 serial dilution; thus, the limit of detection was 107 to 101 copies. Negative control (purified PCR-grade water instead of the DNA template) was included in all PCR reactions.

All participants received dental treatment at University of Taubate Dental School according to individual needs. Periodontally healthy individuals received guidance on oral hygiene and general healthy habits.

### Statistical Analysis

Salivary bacterial counts obtained before and after preoperative mouthwashes were compared in each periodontal status (paired t-test and Student’s t-test). In addition, percentages of reductions were compared among CHX, EO, CPC, and negative control to verify a possible influence of periodontal status on the antimicrobial efficacy of the tested mouthwashes.

The software programmes Bio Estat 5.0 and SPSS 13.0 were used and a statistical significance level of 95% (p < 0.05) was set.

## Results

Table 2 shows demographics, periodontal clinical and microbiological parameters of the study population.

**Table 2:** General characteristics of the study population: demographic data, clinical and microbiological periodontal parameters

Parameter	Periodontal status		
	Health	Gingivitis	Periodontitis
	(n = 60)	(n = 60)	(n = 60)
Age	28.32 ± 8.33	30.19 ± 8.47	46.39 ± 10.21
Female/male	40/20	41/19	34/26
Smokers/never smokers	11/49	05/55	20/40
Plaque index (mean ± SD)	0.41	0.70	1.09
	± 0.13	± 0.20	± 0.11
Gingival index (mean ± SD)	0.62	1.50	1.52
	± 0.14	± 0.40	± 0.18
Periodontal pocket depth in mm (mean ± SD)	1.01	1.40	3.53
	± 0.21	± 0.30	± 0.83
Clinical attachment level in mm (mean ± SD)	0.68	0.96	2.97
	± 0.11	± 0.51	± 0.91
Total bacterial load	130751084.40	110163404.60	115435149.50
*Streptococcus oralis*	3617845.88	21071108.88^a^	3158633.75
*Porphyromonas gingivalis*	32926.86^c^	444219.51^b^	1062176.19^a^
*Tannerella forsythia*	48922.30^c^	154370.46^b^	1134260.24^a^
*Treponema denticola*	37491.38^c^	252868.31^b^	502628.30^a^
Red complex	119340.55^c^	851458.30^b^	2699064.75^a^

Different superscript letters within rows indicate statistically significant differences among products, Student’s t-test (p < 0.05).

The results were described according to the periodontal status group. In the periodontal healthy group, there was a tendency toward reduced total bacterial load, including periodontal pathogens, after rinsing with CPC (total bacterial load, *S. oralis, T. denticola* and *T. forsythia*). This was not the case the negative control group. The mean values before and after the use of preoperative mouthwashes tended to decrease after rinsing with EO (total bacterial load, *S. oralis, T. denticola*). Additionaly, the tendency of reduction was observed using the CHX rinse (*P. gingivalis, T. denticola, T. forsythia*, red complex, total bacterial load) (Table 3).

**Table 3:** Mean bacterial counts observed in periodontally healthy subjects before and after mouthwash

Parameter	Mouthwash				p-value
	Chlorhexidine	Essential oils	Cetylpyridinium chloride	Negative control	
	(n = 15)	(n = 15)	(n = 15)	(n = 15)	
Total bacterial load	77857944.20 ± 185907625.480902^A^	88187978.40 ± 165805763.774564^A^	43933160.60 ± 142224935.5^A^	1381795.13 ± 74777979.68^A^	p > 0.05
*Streptococcus oralis*	1430077.33 ± 6400408.409^B^	3231147.05 ± 13632754.45^B^	258372.15 ± 2617086.7^B^	222706.88 ± 13194695.19^B^	p > 0.05
*Porphyromonas gingivalis*	106326.00 ± 479475.9347^aC^	791.78 ± 2452.803594^bD^	965.37 ± 3815.165463^bC^	0.86 ± 0^bC^	p < 0.05
*Tannerella forsythia*	61557.47 ± 271828.2046^aC^	26695.65 ± 53806.59053^bC^	21106.14 ± 74112.85551^bC^	2199.81 ± 34192.97405^bC^	p < 0.05
*Treponema denticola*	33358.41 ± 133417.2878^aC^	41639.80 ± 102577.6738^aC^	14783.64 ± 49325.32973^bC^	4292.84 ± 53383.00803^bC^	p < 0.05
Red complex	201241.89 ± 882535.3415^aC^	69127.24 ± 133915.3484^bC^	36855.17 ± 122000.3744bC	6493.52 ± 77452.72681^bC^	p < 0.05
	p < 0.05	p < 0.05	p < 0.05	p < 0.05	

Data are shown as separate or combined total bacterial levels. Statistically significant differences between products are indicated as different superscript lowercase letters within rows (paired-t and Student’s t-tests; p < 0.05) and different capital letters within columns.

Similar to the healthy group, in the gingivitis group a tendency toward reduction was also observed after rinsing with CPC (total bacterial load, *S. oralis, T. denticola, T. forsythia*), EO (total bacterial load, *P. gingivalis, S. oralis, T. denticola* and *T. forsythia*) and CHX (total bacterial load and *S. oralis*). The mean values observed showed reductions after rinsing with EO (total bacterial load, *P. gingivalis, T. denticola*, and red complex). Furthermore, CHX reduced *S. oralis*, and CPC was more effective against *T. forsythia* (Table 4).

**Table 4:** Mean bacterial counts observed in subjects with gingivitis before and after mouthwash

Parameter	Mouthwash				p-value
	Chlorhexidine (n = 15)	Essential oils (n = 15)	Cetylpyridinium chloride (n = 15)	Negative control (n = 15)	
Total bacterial load	28058399.33(b)(A) ± 98374149	70588192.29 (a)(A) ± 97910195.85	12172962.23 (b)(A) ± 234738725.1	-19128918.5 (b)(B) ± 158866465.7	p < 0.05
*S. oralis*	78288231.28 (a)(A) ± 295231297.2	1133332.12 (b)(B) ± 4390220.133	2193101.61 (b)(B) ± 10899734.74	-1732402.34 (b)(B) ± 3343444.202	p < 0.05
*P. gingivalis*	-124959.82 (C) ± 62229.15555	316337.27(C) ± 940676.0811	1121.23 (D) ± 4242.234148	77819.45 (A) ± 4699671.521	p>0.05
*T. forsythia*	-1007.63(C) (b) ± 194761.5475	64211.73(D)(b) ± 99822.59976	178571.60 (a)(C) ± 614883.743	-27123.92 (b)(A) ± 174541.9662	p < 0.05
*T. denticola*	78608.26 (b)(B) ± 339072.6136	403680.23 (a)(C) ± 1100177.441	52792.81 (b)(C) ± 175427.8144	170554.07 (b)(B) ± 736969.2264	p < 0.05
Red Complex	- 47359.199 (b)(C) ± 451276.9509	784229.24 (a)(C) ± 1899676.666	232485.66 (b)(C) ± 632185.0201	221249.61 (b)(B) ± 5599207.689	p < 0.05
	p < 0.05	p < 0.05	p < 0.05	p < 0.05	

Data are shown as separate or combined total bacterial levels. Statistically significant differences between products are indicated as different superscript lowercase letters within rows (paired-t and Student’s t-tests; p < 0.05) and different capital letters within columns.

Finally, for the periodontitis group, the total bacterial counts decreased only after rinsing with EO and CHX, but it did not happen with the use of CPC or the negative control. The tendency of reduction of the total bacterial load was maintained after rinsing with CPC (*P. gingivalis, S. oralis,* *T. denticola* and *T. forsythia*), EO (*P. gingivalis, S. oralis,* *T. denticola* and *T. forsythia*) and CHX (*S. oralis, T. denticola* and *T. forsythia*). In terms of reduction of the mean values observed after rinsing, EO was the most effective against total bacterial load, *P. gingivalis, T. forsythia, T. denticola* and red complex. CHX reduced *T. denticola* and *T. forsythia*, and CPC reduced *S. oralis* and *T. denticola* (Table 5).

**Table 5:** Mean bacterial counts observed in subjects with periodontitis before and after mouthwash

Parameter	Periodontitis				p-value
	Chlorhexidine (n = 15)	Essential oils (n = 15)	Cetylpyridinium chloride (n = 15)	Negative control (n = 15)	
Total bacterial load	751807.89 ± 76921279.79^bB^	20072965.34 ± 75704149.81^aA^	-33659256.51 ± 324244444.2^bC^	-18185716.43 ± 127941302^bD^	p < 0.05
*S. oralis*	1143169.86 ± 6414292.642^bA^	1721175.90 ± 3625419.285^bB^	3775319.36 ± 11064348.01^aA^	-1888805.10 ± 5557261.922^bD^	p < 0.05
*P. gingivalis*	57352.88 ± 519934.4661^bC^	985634.33 ± 2663374.737^aB^	777961.05 ± 2896529.363^aB^	11184930.56 ± 4572149.138^bA^	p < 0.05
*T. forsythia*	1226445.53 ± 3009662.259^aA^	1743639.38 ± 6133674.745^aB^	706302.96 ± 1130619.023^bB^	37852.46 ± 292494.1154^bC^	p < 0.05
*T. denticola*	453535.39 ± 1247608.83^aB^	577617.68 ± 1252754.702^aC^	165750.21 ± 414938.1751^bB^	196270.36 ± 789172.2305^bB^	p < 0.05
Red complex	1737333.81 ± 4353635.34^bA^	3306891.40 ± 7587209.081^aB^	1650014.23 ± 3613881.379^bA^	1419053.39 ± 4711277.678^bA^	p < 0.05
	p < 0.05	p < 0.05	p < 0.05	p < 0.05	

Data are shown as separate or combined total bacterial levels. Statistically significant differences between products are indicated as different superscript lowercase letters within rows (paired-t and Student’s t-tests; p < 0.05). and different capital letters within columns.

In all periodontal status groups, bacterial reductions found after preoperative rinses tended to be statistically significant, although with different magnitudes among tested products.

## Discussion

In dental practice, preoperative rinses are employed to counteract oral microbiota as a potential source of infection. In fact, contaminated aerosols and splatter carry the potential for disease transmission to clinicians and patients. Although this specific recommendation for mouthwash use is based on an expected reduction of the risk for local and systemic infections,^2,21,29^ studies designed to identify which factors could influence preoperative rinse efficacy are lacking. Therefore, the present randomised controlled clinical trial investigated whether periodontal status influences the antimicrobial efficacy of a single-dose preoperative mouthwash or not. This seemed plausible since, in the presence of periodontitis, there are higher levels of bacteria and a more complex microbiota which could represent a greater challenge for the antimicrobial action of a mouthwash. As a second aim, the effectiveness of different mouthwashes was compared.

Serban et al^25^ observed a significant positive correlation between the number of decayed teeth and retention of microorganisms in dental masks. Although for a heathy periodontium, CPC was demonstrated to be a reasonable choice as a preoperative mouthwash, for gingivitis and periodontitis, there was a need for more powerful mouthwashes, such as CHX and EO. Data from long-term studies demonstrated that CHX and EO provide greater clinical benefits in comparison to CPC due to CPC’s lower biofilm penetration and substantive property.^6,15,24,30^ However, when a single-dose use is considered, CPC results could be a better option.^10,23,28^ Different variables are related to these differences in outcome, such as type of sample (saliva, biofilm, aerosol), laboratoryl technique (bacterial culture, DNA-DNA hybridization, real-time PCR), active ingredient (CHX, EO, CPC, tea tree oil) and its concentration (0.12% or 0.2% CHX; 0.05, 0.07 or 0.075% CPC), in addition to periodontal status as demonstrated by the present study. The capacity of CHX and EO to penetrate most deeply into biofilm could be responsible for their superior results among diseased patients. Further, due to its faster penetration into biofilm, EO was even better in some circumstances.^6,24^ It is likely that following a sequential analysis, CHX antimicrobial effects would quickly match EO results. This time-related influence had been previously reported by Hunter et al,^16^ who did not identify a statistically significant difference between the CHX preoperative mouthwash group and the no-rinse group. In periodontally diseased patients, Balejo et al^2^ found a distinct reduction in the occurrence of induced bacteremia after rinsing with CHX as the preoperative mouthwash.

Saliva is easily sampled without pain or invasiveness. It is also easily transported and stored, which in clinical trials improves individuals’ compliance.^5^ Besides these advantages, for the present study, saliva was particularly relevant considering its contribution to aerosol contamination. According to Graetz et al,^13^ in the presence of saliva, supragingival scaling is accompanied by greater contamination in comparison to subgingival scaling. In the present study, saliva samples from periodontally healthy or diseased individuals were analysed by a sensitive and reliable molecular technique (real-time PCR).^3^ Up to now, most studies had used bacterial culture^1,11,12,14,22,26,28^ in addition to a few that used qPCR30 or DNA-DNA hybridisation.^10^ Although for the question under investigation, the identification of viable bacteria is of great interest, as some bacterial structures such as LPS (lipopolysaccharides) and even dead bacteria are able to induce a host immune response.^31^ Therefore, molecular quantification is also important.

In this study, in the periodontally healthy group, CPC reduced the total bacterial load counts, demonstrating that it is a good option for clinicians. CHX and EO showed comparable results, but were more effective in comparison to CPC in terms of reducting total bacterial load. In the gingivitis group, EO, CHX, and CPC statistically significantly reduced the total bacterial load, but EO showed better results than the other mouthwashes. A reduction of total bacterial load in the periodontitis group by preoperative EO and CHX mouthwashes was observed, but it did not occur with the CPC rinse.

In the periodontally healthy group, CHX statistically significanlty reduced isolates and the simultaneous presence of *P. gingivalis, T. forsythia*, and *T. denticola*, while EO reduced *S. oralis* and *T. denticola*. CPC was effective for *T. forsythia, T. denticola*, and *S. oralis*. In vitro, it is possible to observe different findings. Simões et al^28^ demonstrated good growth-inhibitory effects for CPC and no inhibitory effects for EO, probably because the latter did not diffuse on agar. Clinically, Albuquerque et al^1^ and Shetty et al^26^ found CHX to be the most effective regarding bacterial reductions although periodontal status was not considered.

In the gingivitis group, EO provided the greatest reductions of total bacterial load and Gram-negative bacteria (*P. gingivalis, T. denticola*, and red complex species). CHX more greatly reduced the Gram-positive species, while CPC was superior in the reduction of *T. forsythia*. Without taking into consideration periodontal condition, Fine et al^11^ reported in two studies acceptable efficacy of EO as a preoperative mouthwash. The individuals of the two studies received ultra-sonic dental prophylaxis for 10 min; one group used a preoperative mouthwash with EO and the other used a control rinse. Samples of the aerosol were collected in sterile filters immediately after the end of the treatment in the first study, and 40 min after the end of the treatment in the second study. The results of the study concluded that the use of a preoperative mouthwash containing an antimicrobial agent can reduce cross-infection in dental surgery.

Finally, among individuals with periodontitis, the most significant reductions of total bacterial load, *P. gingivalis*, and red complex species were observed after EO use. *Tannerella forsythia* and *Treponema denticola* decreased to similar extents after the use of CHX and EO, also CPC reduced the Gram-positive species. Also among individuals with periodontitis, Gupta et al^14^ compared CHX, an herbal solution, and water. Preoperative rinses with CHX or herbal solutions eliminated most bacteria in aerosols, with more significant reductions in the first rinse.

Considering differences in the microbiota, to reach a statistically significant bacterial reduction in the presence of periodontitis, the mouthwash should be more effective than in the presence of periodontal health.

Thus, in the present study, the preoperative mouthwashes tested presented different degrees of efficacy, and it is certain that periodontal status guides the choice of rinse by clinicians, who must integrate them into their therapeutic scheme as part of biosafety procedures.

## Conclusions

Periodontal status influenced the antimicrobial efficacy of preoperative mouthwashes. Antimicrobial effects differed among mouthwashes, with CHX and EO showing the greatest efficacy. Therefore, periodontal status should be taken into consideration by clinicians. For periodontally healthy individuals, CHX, EO, or CPC could be used as preoperative mouthwashes; however, for periodontally diseased patients, EO and CHX are the most effective. The present study confirmed the clinical recommendation of CHX as a preoperative mouthwash, and that EO is a similarly effective option for daily practice.

Information related to the antimicrobial efficacy of preoperative mouthwashes according to periodontal status is scarce. Our study showed that the recognition of periodontal condition by clinicians is mandatory to select the most effective preoperative mouthwash.
